# Within-Sulfonylurea-Class Evaluation of Time to Intensification with Insulin (ZODIAC-43)

**DOI:** 10.1371/journal.pone.0157668

**Published:** 2016-06-21

**Authors:** Dennis Schrijnders, Laura C. Hartog, Nanne Kleefstra, Klaas H. Groenier, Gijs W. D. Landman, Henk J. G. Bilo

**Affiliations:** 1 Diabetes Centre, Isala, Zwolle, the Netherlands; 2 Langerhans Medical Research Group, Zwolle, the Netherlands; 3 University of Groningen, University Medical Center Groningen, Department of Internal Medicine, Groningen, the Netherlands; 4 University of Groningen, University Medical Center Groningen, Department of General Practice, Groningen, the Netherlands; 5 Department of Internal Medicine, Gelre Hospital, Apeldoorn, the Netherlands; 6 Department of Internal Medicine, Isala, Zwolle, the Netherlands; Weill Cornell Medical College Qatar, QATAR

## Abstract

**Background:**

Previous studies have shown that many within-class differences exist between sulfonylureas (SUs), however, whether differences exist regarding the time it takes between initiating an SU and the need to intensify treatment with insulin is unclear. The aim of this study was investigate the relationships between the three frequently used sulphonylureas, prescribed as dual therapy next to metformin, and the time needed to treatment intensification with either insulin or oral triple therapy in patients with type 2 diabetes mellitus.

**Methods:**

Zwolle Outpatient Diabetes project Integrating Available Care (ZODIAC) is a prospective observational cohort study set in primary care in the Netherlands. Annually collected data on diabetes medication and clinical variables within ZODIAC are used to evaluate the primary outcome, time to insulin and secondary outcome, time to either insulin or triple oral therapy. For statistical analysis a time-dependent cox proportional hazard model was used.

**Results:**

3507 patients were included in the analysis, with a mean age of 61 (SD 11.4) and a median HbA1c of 6.8% [IQR 6.4–7.4] (50.8 mmol/mol [IQR 46.4–57.4]).The hazard ratio (HR) for the primary endpoint was 1.10 (95% CI 0.78–1.54) for metformin/glimepiride and 0.93 (95% CI 0.67–1.30) for metformin/tolbutamide with metformin/gliclazide as reference group. The HR for the secondary outcome was 1.04 (95% CI 0.78–1.40) and 0.85 (95% CI 0.64–1.13), respectively.

**Conclusion:**

In this large Dutch primary care cohort, new users of neither gliclazide, glimepiride nor tolbutamide as dual therapy with metformin, resulted in differences in the time needed for further treatment intensification.

## Introduction

Many important within-class SU differences have been described, mostly in favour of gliclazide. For example; gliclazide is considered the safest SU in patients with renal impairment[1]. Furthermore, there is also a clear benefit of prescribing gliclazide for reasons of hypoglycaemia risk[2] and possibly cardiovascular safety[3]. Within-class SU differences have also been described for SU failure rate. For example, glipizide and glibenclamide are associated with a higher failure rate compared to gliclazide[4]. In the 2013 Dutch type 2 diabetes mellitus (T2DM) guideline, gliclazide specifically became the preferred sulphonylurea (SU) and the first intensification step after metformin[5].

When dual oral therapy fails, next to lifestyle interventions, a switch to or addition of, once daily insulin or possibly a third oral agent are advised steps for regaining adequate glycaemic control. This moment in time could be considered a turning point for patients and is regarded as disease progression for patients and therefore is a relevant surrogate endpoint.

Whether within-class differences exist for “time needed to intensification” for SUs remains unclear. We hypothesized that a difference in time to intensification could be present, since the subsequent generations of SUs have become increasingly more potent[4,6]. The presence of a within-class difference concerning time to insulin could benefit both patient related outcomes as well as aid in controlling health care costs since treatment with oral glucose lowering agents is more cost-effective than treatment with insulin[7].

Using a new-user design, the aim of this study was to investigate the relationships between the three most frequently prescribed SUs in the Netherlands, prescribed as dual therapy next to metformin and the time needed to treatment intensification with either insulin or oral triple therapy in patients with T2DM.

## Methods

The study is reported according to the STROBE (Strengthening the reporting of observational studies in epidemiology) recommendations[8].

### Study design and data collection

This study is part of the prospective ZODIAC (Zwolle Outpatient Diabetes project Integrating Available Care) cohort study. This ongoing study started in 1998 in the Zwolle region, and has since expanded to more than 600 general practices in the north-east and western part of the Netherlands. Following two major expansions of the cohort in 2006 and 2009, the majority of patients were included from 2006 onwards. Patients included in the study are diagnosed with T2DM and are exclusively treated in primary care. Data are collected annually by general practitioners and send to the diabetes centre annually.

### Patient selection

A “new-user” design[9] was used. Patients who used metformin monotherapy for at least one year and subsequently intensified for the first time with gliclazide, glimepiride or tolbutamide were selected. Patients were also required to be included in the ZODIAC study between 1998 and 2012. Patients with an eGFR below 30mL/min/1.73m^2^ or when eGFR at baseline was missing were excluded. We hypothesized that including these patients could lead to selection bias because gliclazide is the preferred SU in renal impairment[1].

### Outcome measures

The primary outcome was the first receipt of insulin and secondary outcome was first receipt of either insulin or triple oral therapy.

### Statistical analysis

Quantitative variables are presented as means with standard deviation for normally distributed values, and median and interquartile range (IQR) for skewed variables. Time-dependent cox proportional hazard analyses, adjusted for HbA1c, age, gender, diabetes duration, updated mean creatinine and updated mean BMI were used to evaluate the primary and secondary outcome. All analyses were performed with IBM SPSS Statistics (Version 22.0).

### Ethics statement

In the ZODIAC study, patients consented to anonymous use of their data for study purposes. The medical ethics committee of Isala, Zwolle, The Netherlands approved the ZODIAC study (METC reference numbers 03.0316 and 07.0335).

## Results

**Fig 1:**
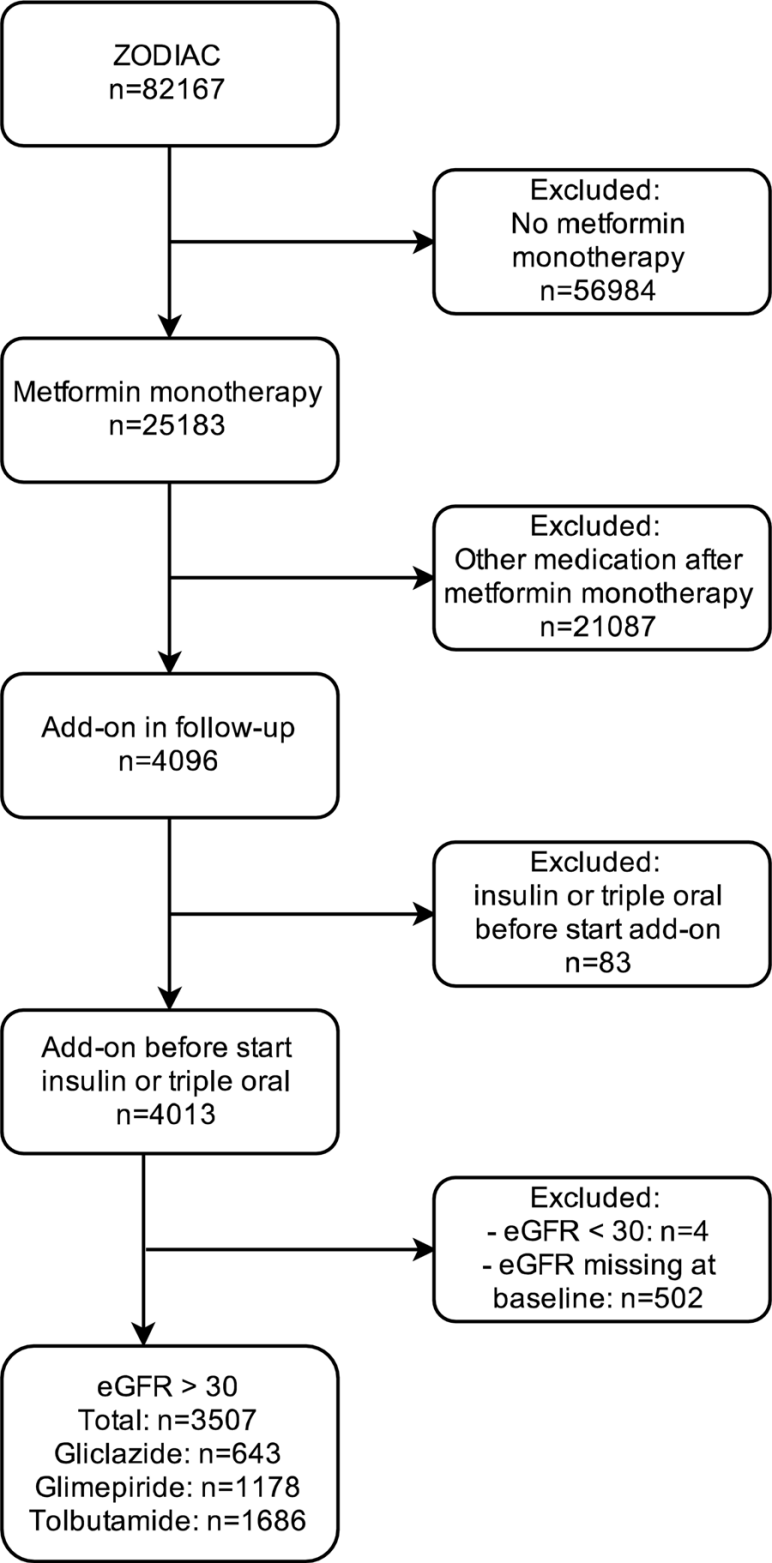
Flow of selected patients.

From the complete cohort (N = 82.167), 25.183 patients used metformin monotherapy. From these, 4096 received some form of intensification. Patients receiving insulin as second step on top of metformin (n = 83) and 506 patients with an eGFR < 30 ml/min/1.73m2 or missing eGFR were excluded (Fig 1). From the 3507 selected patients, 47% was female, the mean age was 61 (SD 11.4), median HbA1c 6.8% [IQR 6.4–7.4] (50.8 mmol/mol [IQR 46.4–57.4]), median BMI 29.7 [IQR 26.8–33.3], median diabetes duration 6.8 years [IQR 4.5–9.4], mean eGFR 83.2 (SD 20.1) and median creatinine 76.0 [IQR 65.0–89.0] (Table 1). There were no baseline differences in median HbA1c between the different treatment groups; metformin/gliclazide 6.9 [IQR 6.4–7.4], metformin/glimepiride 6.8 [IQR 6.3–7.4] and metformin/tolbutamide 6.9 [IQR 6.4–7.5]. Two-and-half and 5 years after intensification 13.0% and 32.0% of patients were using insulin, respectively. Two-and-half and 5 years after intensification 17.5% and 39.7% of patients were using either insulin or triple oral therapy, respectively.

**Table 1:** Baseline characteristics.

Number of patients	3507
Gender^a^	Female: 47%, Male: 53%
Age at diagnosis (years)^b^	57.8 [17.9]
Age (years)^b^	61.0 [11.4]
eGFR^b^	83.2 [20.1]
Creatinine (μmol/L)^c^	76.0 [65.0–89.0]
Diabetes duration (years)^c^	6.8 [4.5–9.4]
HbA1c (%) / HbA1c (mmol/mol) ^c^	6.8 [6.4–7.4] / 50.8 [46.4–57.4]
BMI^c^	29.7 [26.8–33.3]

^a^%

^b^ mean with SD

^c^ median with interquartile range

### Primary endpoint

**Fig 2:**
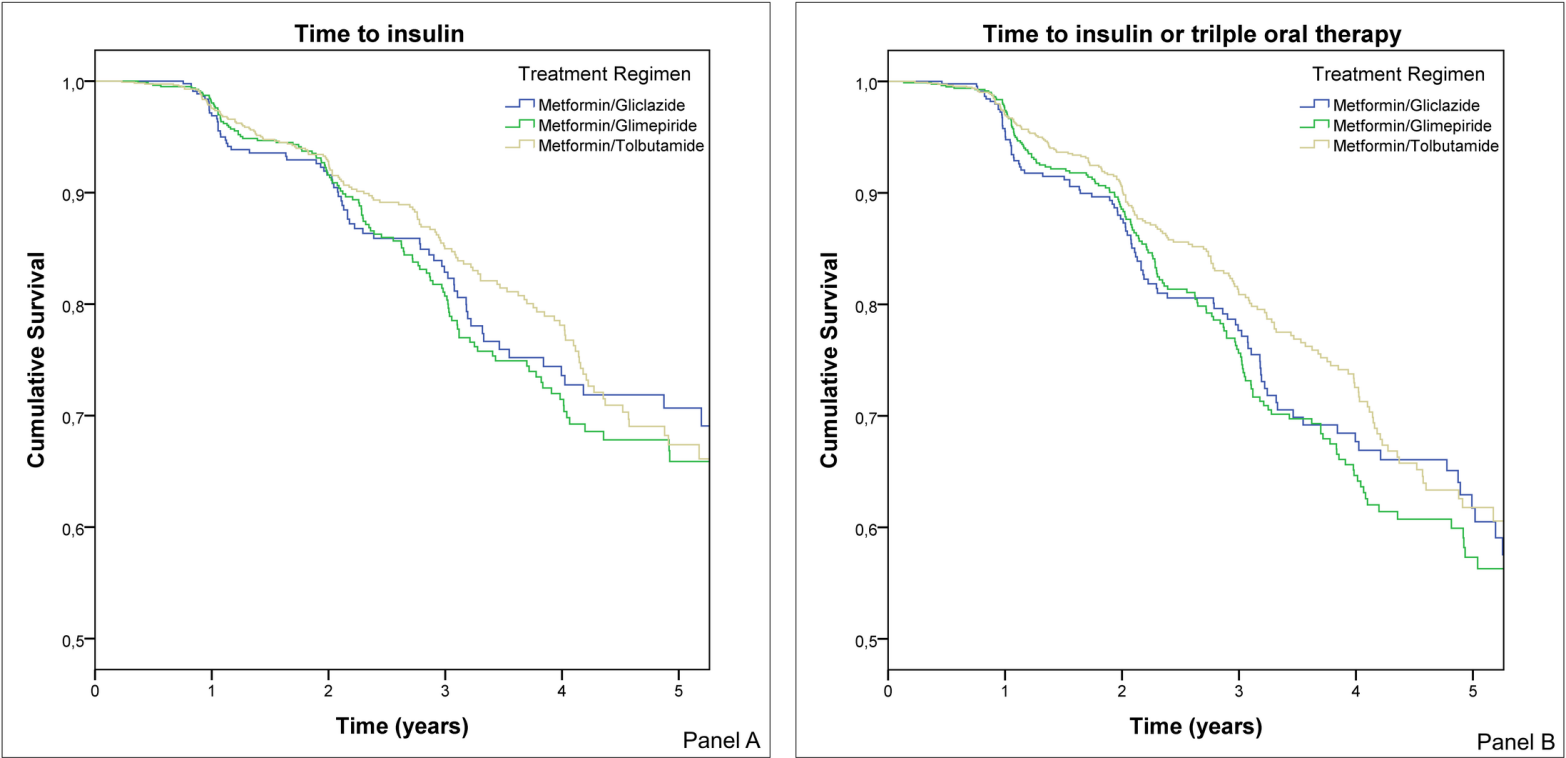
Cumulative survival. Panel A shows the cumulative survival for gliclazide, glimepiride and tolbutamide and time to insulin using the unadjusted model. Panel B shows the cumulative survival for gliclazide, glimepiride and tolbutamide and time to insulin or triple oral therapy using the unadjusted model.

In the unadjusted model the HR for metformin/glimepiride was 1.15 (95% CI 0.83–1.61) and for metformin/tolbutamide 0.97 (95% CI 0.70–1.34), with metformin/gliclazide as a reference category. There were no significant differences between the three groups in primary outcome (Fig 2). The pairwise HR's for the primary endpoint in the unadjusted model are shown in Table 2.

**Table 2:** Unadjusted model.

	Time to insulin			Time to insulin or third oral agent		
	HR	95% CI	p	HR	95% CI	p
Medication			0.429			0.161
Gliclazide* vs Glimepiride	1.151	0.825–1.605	0.407	1.111	0.834–1.480	0.470
Glicazide* vs Tolbutamide	0.965	0.695–1.340	0.831	0.878	0.660–1.168	0.371
Glimepiride* vs Tolbutamide	0.838	0.637–1.103	0.207	0.790	0.620–1.006	0.056

*Reference category.

The HR for time to insulin using a fully corrected model for metformin/glimepiride was 1.10 (95% CI 0.78–1.54) and for metformin/tolbutamide 0.93 (95% CI 0.67–1.30), with metformin/gliclazide as a reference category. The pairwise HR's for the primary endpoint in the fully corrected model are shown in Table 3. There were no significant differences between the three groups in primary outcome. HbA1c and age are significantly associated with the primary endpoint with HRs of 1.08 (95%CI 1.00–1.15) and 0.99 (95%CI 0.97–1.00) respectively. Creatinine, BMI, gender, and diabetes duration are not significantly associated with the primary endpoint (Table 3).

**Table 3:** Covariates included in Cox proportional hazard model.

	Time to insulin			Time to insulin or third oral agent		
	HR	95% CI	p	HR	95% CI	p
HbA1c	1.077	1.008–1.151	0.028	1.082	1.022–1.145	0.007
Creatinine	0.998	0.990–1.006	0.688	0.992	0.985–1.000	0.410
BMI	1.007	0.983–1.033	0.585	1.021	1.000–1.043	0.510
Gender	1.012	0.768–1.334	0.934	0.896	0.701–1.145	0.380
Diabetes duration	0.988	0.951–1.026	0.545	0.994	0.969–1.020	0.644
Age	0.986	0.974–0.998	0.026	0.984	0.974–0.995	0.003
Medication			0.541			0.234
Gliclazide* vs Glimepiride	1.095	0.780–0.537	0.602	1.044	0.780–1.397	0.772
Glicazide* vs Tolbutamide	0.933	0.668–1.304	0.684	0.848	0.635–1.134	0.266
Glimepiride* vs Tolbutamide	0.852	0.643–1.131	0.268	0.813	0.634–1.041	0.101

* reference category

### Secondary endpoint

In the unadjusted model the HR for metformin/glimepiride was 1.11 (95% CI 0.83–1.48) and for metformin/tolbutamide 0.88 (95% CI 0.66–1.17), with metformin/gliclazide as a reference category. There were no significant differences between the three groups in primary outcome (Fig 2). The pairwise HR's for the primary endpoint in the unadjusted model are shown in Table 2.

The HRs for time to insulin or triple oral therapy for metformin/glimepiride was 1.04 (95% CI 0.78–1.40) and for metformin/tolbutamide 0.85 (95% CI 0.64–1.13) with metformin/gliclazide as a reference category. The pairwise HR's for the secondary endpoint are shown in Table 3. HbA1c (HR 1.08, 95% CI 1.02–1.15) and age (HR 0.98, 95% CI 0.97–1.00) were significantly associated with the need for intensification therapy with insulin of a third oral agent. Risk for time to insulin or triple oral therapy by increased 8% per 1% increase in HbA1c and decreased 2% per 1 year increase in age. Creatinine, BMI, Gender and diabetes duration were not significantly associated with the secondary endpoint (Table 3).

## Discussion

In this primary care cohort of patients who used metformin monotherapy and intensified with gliclazide, glimepiride or tolbutamide, the so-called new users, there were no statistically significant differences in time to insulin, or time to either insulin or a third oral agent. After 2.5 and 5 years respectively 13.0% and 32.0% of patients were using insulin and respectively 17.5% and 39.7% of patients were using insulin or triple therapy. The numbers are in accordance with the numbers found previous studies[10].

No studies were found investigating within-class differences in time to insulin in patients using metformin and a SU as dual therapy. A retrospective cohort study conducted in Colombia showed a higher risk of insulin treatment (OR = 3.49, 95%CI 2.61–4.66) in patients treated with metformin and glibenclamide[11]. In a Swedish retrospective cohort study the probability of insulin use was increased (HR = 2.71, 95%CI: 2.15–3.43) in patients using more than one oral antidiabetic agent[12]. However, the risk of insulin use was not evaluated in individual agents within the SU class.

Based on the results of this study, that in daily primary care practice, the time to intensification with insulin or a third oral agent is not influenced by the choice of a specific SU but rather by specific patient characteristics despite the differences in potency between the three SUs[4,6]. In this study, HbA1c and age appeared to be more important factors.

The strengths of this study were the inclusion of patients from a daily care setting and the new-user design. The daily care setting increases the generalizability of our results. Excluding prevalent users allowed us to avoid underascertainment of insulin prescription that occurs early after the start of SUs and also allowed us to avoid that confounders are influenced by previous treatment[9]. A limitation was that the data is collected annually; changes in medication within this year might not be reflected in our database. A second limitation is the possibility that patients who required insulin could have been referred to secondary care. Patients within ZODIAC are lost to follow-up when referred to secondary care for their diabetes treatment. However, in the Netherlands insulin treatment is usually initiated in primary care and thus expect the underestimation of time to insulin to be limited. Furthermore, patients could have refused intensification of therapy like the addition of insulin, leading to an underestimation of the primary endpoint which was the reason for also analysing the time needed to a third oral agent. However, we don’t expect a between-group difference in motivation to use insulin.

## Conclusion

In conclusion, there were no differences found in this large Dutch primary care cohort with respect to the time needed for further treatment intensification with neither insulin nor a third oral agent when treated with gliclazide, glimepiride or tolbutamide on top of metformin. In this study higher HbA1c and lower age are associated with a shorter time needed to insulin and a shorter time to a third oral agent.

## Supporting Information

S1 File STROBE Checklist. (DOCX)

S2 File SPSS Output files. (ZIP)
